# Perforated gastric ulcer triggering acute cholecystitis: Case report of an uncommon and deceptive presentation

**DOI:** 10.1016/j.ijscr.2025.111723

**Published:** 2025-07-23

**Authors:** Brandon Velazquez, Madison Magee, Daniel Dove, Benetta Miller, Zbigniew Moszczynski

**Affiliations:** a Bayonne Medical Center, 29 E 29th St, Bayonne, NJ 07002, USA

**Keywords:** Case report, Gastric ulcer perforation, Peptic ulcer-induced cholecystitis, Misdiagnosed perforated ulcer, Acute cholecystitis, Occult peptic ulcer disease

## Abstract

**Introduction:**

Perforated peptic ulcer disease (PUD) is a critical condition that can present with atypical symptoms, leading to misdiagnosis and treatment delays. While PUD classically presents with peritonitis, rare cases may induce localized inflammatory responses, mimicking other abdominal pathologies such as acute cholecystitis.

**Presentation of case:**

We report the case of a 29-year-old male with recurrent postprandial right upper quadrant (RUQ) and epigastric pain. Despite multiple emergency department (ED) visits, normal initial imaging led to misdiagnoses of gastroenteritis and gastroesophageal reflux disease (GERD) with cannabis-induced hyperemesis syndrome. On his third ED visit, he was diagnosed with a perforated gastric ulcer complicated by acute cholecystitis, requiring urgent surgical intervention.

**Discussion:**

This case highlights the diagnostic challenges associated with atypical presentations of perforated PUD. The absence of gallstones and initial negative imaging studies contributed to diagnostic delays. Advanced imaging, including computed tomography (CT), plays a crucial role in detecting subtle signs of perforation. Additionally, the inflammatory interaction between the gastric and hepatobiliary systems underscores the need for clinicians to consider ulcer-related complications when evaluating persistent epigastric and RUQ pain.

**Conclusion:**

A high index of suspicion is essential when assessing patients with recurrent abdominal pain despite unremarkable initial evaluations. Early recognition and appropriate imaging can facilitate timely intervention, reducing morbidity associated with delayed diagnosis of perforated PUD and its complications.

## Highlights

• Gastric ulcer perforation mimicking acalculous cholecystitis caused diagnostic delay.
• Ultrasound missed early perforation, highlighting need for advanced imaging.
• Laparoscopy revealed a 3 cm gastric perforation hidden by adhesions.
• Cholecystectomy and Graham patch repair achieved successful recovery.

## 1 Introduction

Peptic ulcer disease (PUD) is a common gastrointestinal condition, but its complications can present deceptively, leading to diagnostic challenges. One of the most serious complications is perforation, typically manifesting as acute peritonitis. However, in rare cases, a perforated gastric ulcer can induce inflammation in adjacent organs, mimicking other acute abdominal pathologies.

Acute cholecystitis is usually associated with gallstones, but acalculous cholecystitis can occur in critically ill patients or as a response to systemic inflammation. When a perforated gastric ulcer induces gallbladder inflammation, the clinical picture can mislead clinicians, resulting in misdiagnoses and delays in management.

Gastric ulcers result from breakdown of the mucosal barrier, usually exceeding 5 mm in diameter, with *H. pylori* infection and nonsteroidal anti-inflammatory drugs (NSAID) use being common causes. Less frequent causes include Zollinger-Ellison syndrome, infections (e.g., CMV), malignancy, Crohn's disease, and smoking [1,2].

This case report describes the case of a 29-year-old male who experienced multiple emergency department (ED) visits for postprandial epigastric pain and ultrasound findings of gallbladder edema without gallstones. Initially misdiagnosed with gastroenteritis, GERD, and cannabis-induced hyperemesis syndrome, he eventually was found to have a perforated gastric ulcer with associated acute cholecystitis. Atypical cases of ulcer disease may be overlooked in younger patients with normal imaging. Greater awareness is needed to ensure early diagnosis in such scenarios [3].

The work presented here has been reported in line with the SCARE criteria [4].

## 2 Presentation of case

A 29-year-old male with no significant medical history aside from daily cannabis use presented with a two-week history of sudden-onset, sharp, burning epigastric pain associated with nausea and vomiting. Pain was postprandial with spontaneous partial resolution. He reported a 30-pound weight loss over two months due to postprandial epigastric pain. Self-medication with 900 mg of ibuprofen exacerbated his pain, prompting presentation to the emergency department. He denied bowel habit changes, fever, or systemic symptoms. He denied a history of diabetes mellitus, HIV infection, or chronic corticosteroid use.

On examination, he was alert and oriented. Laboratory studies showed no leukocytosis, with normal liver function tests, bilirubin, and lipase. Physical examination revealed severe epigastric and right upper quadrant (RUQ) tenderness, a positive Murphy sign, and voluntary guarding. There were no signs of peritonitis such as rigidity or rebound tenderness.

A RUQ ultrasound showed biliary sludge, gallbladder wall thickening up to 7 mm, and pericholecystic edema but no gallstones (Fig. 2). A CT of the abdomen and pelvis with contrast demonstrated an under-distended gallbladder with pericholecystic fluid and edema, along with mild mucosal enhancement and bowel wall thickening of the adjacent duodenum. The pancreas, spleen and adrenal glands were unremarkable, and no free intraperitoneal air was identified (Fig. 3).

**Fig. 1:**
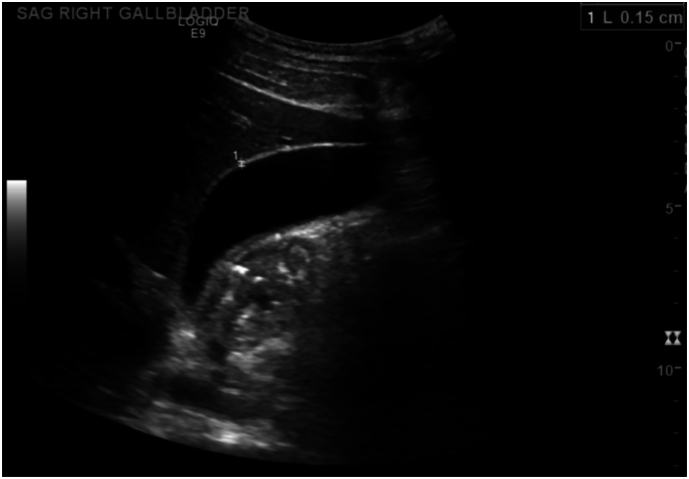
Abdominal ultrasound showing a normal gallbladder wall with absence of gallstones and pericholecystic fluid.

The patient had visited the ED twice previously over two weeks for similar, milder symptoms. Earlier ultrasound studies demonstrated normal gallbladder wall thickness of 1.5 mm (Fig. 1). At that time, the patient was diagnosed with presumed gastroenteritis, followed by a provisional diagnosis of gastroesophageal reflux disease (GERD) and cannabinoid hyperemesis syndrome.

**Fig. 2:**
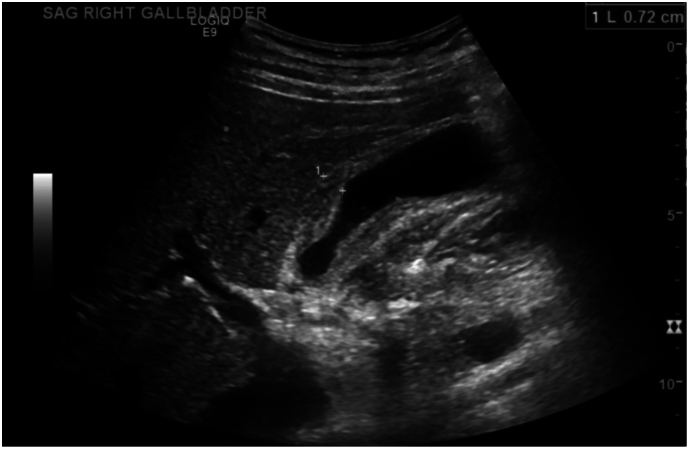
Abdominal ultrasound showing a thickened gallbladder wall with pericholecystic fluid.

**Fig. 3:**
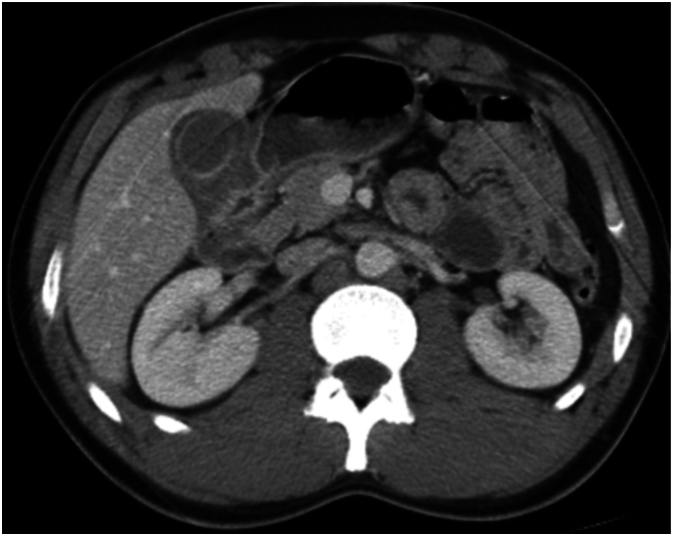
Computed tomography showing a thickened gallbladder wall, a significant amount of pericholecystic fluid, and thickening of gastric and duodenal walls.

**Fig. 4:**
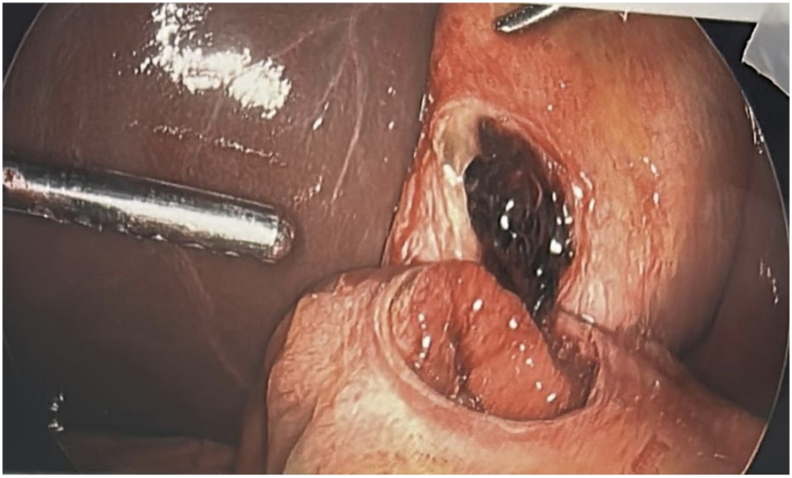
Intraoperative findings: 3 cm perforated ulcer in anterior stomach fistulizing into the gallbladder.

**Fig. 5:**
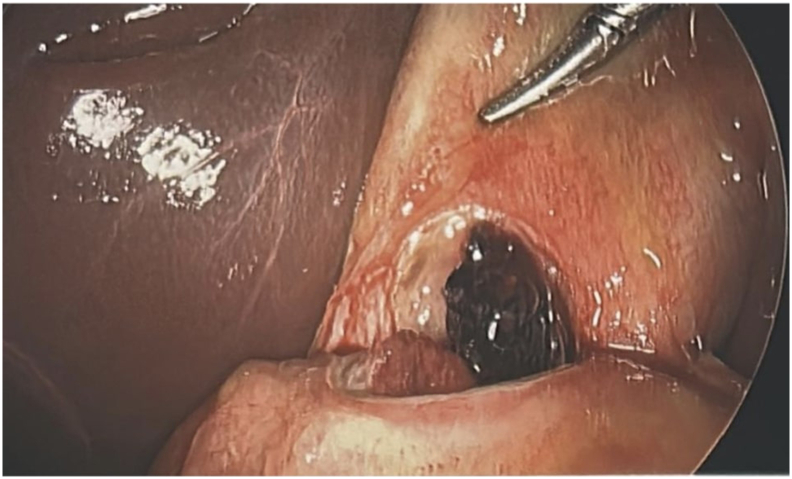
Intraoperative findings: 3 cm perforated ulcer in anterior stomach fistulizing into the gallbladder.

**Fig. 6:**
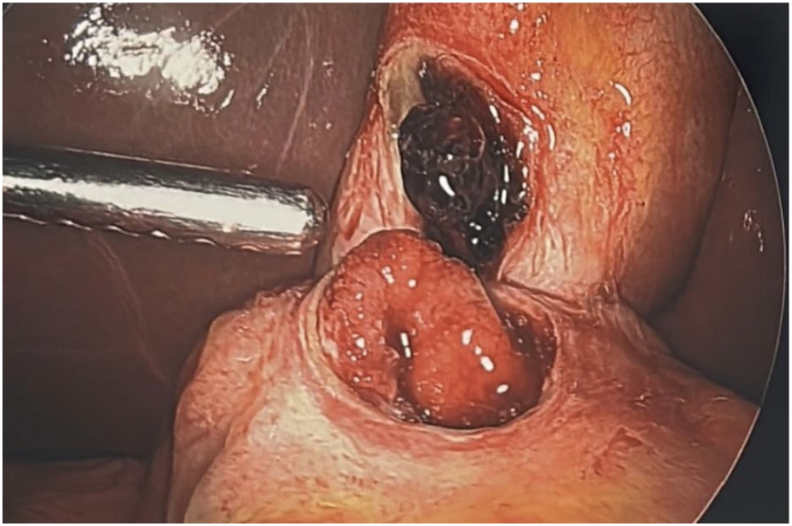
Intraoperative findings: 3 cm perforated ulcer in anterior stomach fistulizing into the gallbladder.

**Fig. 7:**
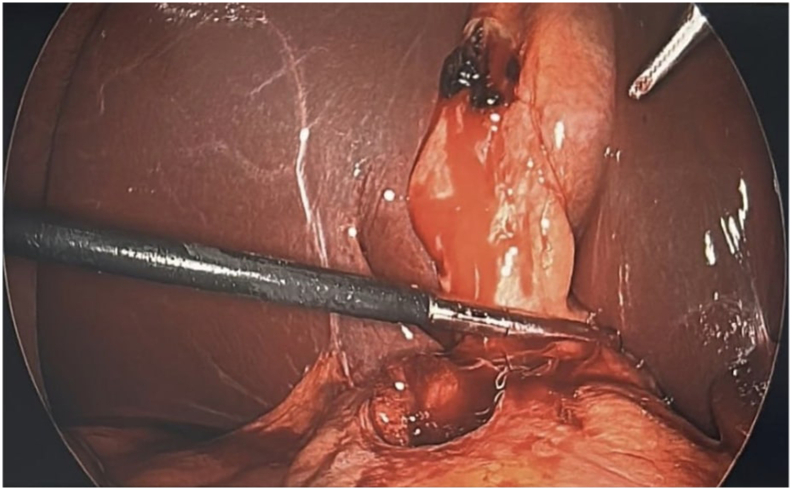
Intraoperative findings: 3 cm perforated ulcer in anterior stomach fistulizing into the gallbladder.

Based on examination and imaging, acute cholecystitis secondary to biliary sludge was diagnosed, and laparoscopic cholecystectomy was planned after initiating IV fluids and antibiotics.

During surgery, a supraumbilical 10 mm port was placed using the Optiview technique. The gallbladder appeared distended, and bile was aspirated. An adhesion between the anterior stomach and the gallbladder fundus was noted. Gentle dissection revealed a 3-cm perforation of the anterior gastric wall (Fig. 4, Fig. 5, Fig. 6, Fig. 7). Given the prolonged symptoms, it was suspected that the perforation had been concealed by the gallbladder. An initial attempt was made to complete the Graham patch repair laparoscopically, but there was difficulty obtaining adequate visualization and exposure of the area. Following gallbladder mobilization and removal, a vertical midline incision was made. Moderate bilious fluid was encountered. Inspection revealed an anterior gastric perforation proximal to the pylorus characterized by smooth edges and mild fibrinous exudate, favoring an acute inflammatory ulcer causing secondary acute cholecystitis, rather than a chronic fibrous fistulous tract. The perforation was repaired using a modified Graham patch with interrupted 2-0 vicryl sutures. A 19F Blake drain was placed near the repair site. Pathology confirmed acute cholecystitis without gallstones and a gallbladder wall thickness up to 9 mm.

Postoperatively, the patient's course was uneventful. The nasogastric tube was removed after two days once normal bowel function resumed. The Blake drain output decreased by postoperative day five, and the patient was discharged on proton pump inhibitors and counseled on avoidance of NSAIDs.

A week later, the patient returned with epigastric pain and vomiting. A CT abdomen and pelvis with oral contrast ruled out obstruction or postoperative collection. Symptoms were worsened by lying down, suggesting bile or acid reflux. A trial of sucralfate provided symptom relief, and he was discharged with outpatient gastroenterology follow-up.

## 3 Discussion

Perforated PUD is a life-threatening condition that can present atypically, delaying diagnosis and management. Classic presentation involves sudden severe abdominal pain and peritonitis, but atypical cases, such as a perforated ulcer causing cholecystitis, are rare and require heightened awareness.

Our patient presented with postprandial epigastric and RUQ pain, initially diagnosed as gastroenteritis, GERD, and cannabis-induced hyperemesis due to normal imaging findings. Notably, no abdominal X-ray was obtained during the initial emergency department evaluations. Surgical consultation was obtained only after ultrasound and CT imaging were performed. Earlier acquisition of plain abdominal radiographs may have demonstrated pneumoperitoneum—an indirect but classical radiographic finding of a perforated peptic ulcer—and thereby facilitated more timely surgical evaluation [6,7]. Moreover, despite the patient's recurrent symptoms of heartburn and a differential diagnosis that included gastroesophageal reflux disease (GERD) and cannabinoid hyperemesis syndrome, an upper endoscopy (EGD) was never performed prior to the acute surgical presentation. Endoscopic visualization of the gastric mucosa remains the gold standard for diagnosing peptic ulcers and could have directly identified the lesion, thereby significantly altering the diagnostic and management trajectory [1,3].

Peptic ulcer perforation allows gastric contents to leak into the peritoneal cavity, leading to chemical peritonitis or localized inflammation. This case highlights how such inflammation can extend to adjacent organs such as the gallbladder, contributing to acalculous cholecystitis [5]. The greater omentum's role in walling off intra-abdominal infections can also obscure classic findings, further complicating diagnosis [8,9].

The possibility that the initial pathology was in the gallbladder, with formation of a cholecystogastric fistula secondarily leading to gastric perforation, should also be considered in similar clinical presentations. However, in this case, that sequence is highly unlikely. Intraoperatively, there was no evidence of a fistulous tract between the gallbladder and the stomach. The gallbladder was mobilized without difficulty and removed without disruption of any gastric tissue or communication with the gastric lumen. In contrast, a distinct 3 cm anterior gastric perforation was clearly identified proximal to the pylorus, which was repaired using a modified Graham patch. Pathological analysis of the gallbladder revealed acute inflammation without gallstones, chronic changes, or signs suggestive of a fistula. Furthermore, preoperative imaging did not show pneumobilia or other hallmark features of a cholecystoenteric fistula [6,10]. Taken together, these findings support that the primary pathology was a perforated gastric ulcer, with secondary localized inflammation of the adjacent gallbladder rather than a fistula-driven process.

Accurate diagnosis requires imaging beyond ultrasound. While ultrasound may miss early ulcer perforations, CT is superior in identifying indirect signs like pneumoperitoneum [6,10]. Our case reinforces the importance of CT in patients with persistent or worsening symptoms despite unremarkable ultrasound findings.

Standard management for perforated PUD involves surgical repair, typically with an omental patch, while cholecystectomy remains the choice for acute cholecystitis [11,12]. In this patient, surgical intervention addressed both pathologies simultaneously.

The concern for malignancy was low on our differential diagnosis, given the patient's recurrent presentations with epigastric pain, nausea, and vomiting, as well as the ulcer's appearance characterized by smooth edges and mild fibrinous exudate. The lesion's location further supported the likelihood of a contained perforated benign ulcer. Although concern for malignancy was low based on intraoperative findings, biopsy at the time of repair would have been prudent. Postoperatively, the patient was evaluated by gastroenterology, and an *H. pylori* stool antigen test was ordered; however, the patient did not have a bowel movement prior to discharge. An outpatient upper gastrointestinal endoscopy (EGD) was scheduled to confirm ulcer healing, assess for possible malignancy, and perform *Helicobacter pylori* testing to guide eradication therapy. Preventing recurrence of peptic ulcer disease requires identifying risk factors such as *H. pylori* infection, NSAID use, and hypersecretory conditions like Zollinger-Ellison syndrome, with appropriate eradication therapy and lifestyle modifications as essential components of comprehensive management [[1], [2], [3]].

This case illustrates the importance of maintaining a broad differential when evaluating patients with recurrent RUQ and epigastric pain, particularly when initial imaging is non-diagnostic. Clinicians should be alert to rare complications such as ulcer-induced cholecystitis to avoid misdiagnosis and ensure timely surgical management. Further research into inflammatory interactions between gastric and hepatobiliary structures may enhance understanding and improve care.

## 4 Conclusion

This case highlights the diagnostic difficulties posed by atypical presentations of perforated peptic ulcer disease. Persistent RUQ and epigastric pain with normal initial imaging should prompt consideration of alternative diagnoses.

A high index of suspicion, early advanced imaging, and timely surgical intervention are vital to prevent complications. Clinicians must remain vigilant for rare conditions like ulcer-induced cholecystitis to enable prompt and effective management.

## Author contribution

- Brandon Velazquez, MD: Study concept or design, data collection, data analysis or interpretation, writing the paper, final review, post submission edits.

- Madison Magee: Data collection, data analysis or interpretation, writing the paper.

- Daniel Dove, MD: Study concept or design, data analysis or interpretation.

- Benetta Miller, MD, FACS: Study concept or design, data analysis or interpretation, final review.

- Zbigniew Moszczynski, MD, FACS: final review, Program Director of Carepoint Health General Surgery Residency.

## Consent

Written informed consent was obtained from the patient for publication of this case report and accompanying images. A copy of the written consent is available for review by the Editor-in-Chief of this journal on request.

## Ethical approval

Ethics approval is not required for case reports at this institution, Bayonne Medical Center.

## Guarantor

-Brandon Velazquez, MD.

-Daniel Dove, MD.

-Benetta Miller, MD.

## Research registration number

Not applicable.

## Funding

There were no external sources of funding for the elaboration of this article.

## Conflict of interest statement

The authors declare no conflicts of interest regarding this case report.
